# Lung capacity is a determinant of cardiovascular disease and myocardial infarction

**DOI:** 10.1186/s12931-026-03502-y

**Published:** 2026-01-13

**Authors:** Ben Knox-Brown, Jean Pierre Sibomana, Karl P Sylvester, Andre F.S. Amaral

**Affiliations:** 1 Cambridge University Hospitals NHS Foundation Trust, Cambridge, United Kingdom; 2 Royal Papworth Hospital NHS Foundation Trust, Cambridge, United Kingdom; 3 National Heart and Lung Institute, Imperial College London, London, UK; 4 Umurinzi Petros Medical Centre, Muhanga, Rwanda; 5 King Faisal Hospital Rwanda, University of Rwanda, Kigali, Rwanda; 6 NIHR Imperial Biomedical Research Centre, London, UK

**Keywords:** Clinical epidemiology, Cardiovascular disease, Lung function

## Abstract

**Introduction:**

There is growing evidence suggesting that lung capacity is associated with risk of cardiometabolic disease. However, most studies rely on spirometric measures of lung capacity and self-reported cardiometabolic disease. We aimed to investigate the association of total lung capacity (TLC) with cardiometabolic disease defined using ICD-10 codes.

**Methods:**

Data from adult patients referred to Cambridge University Hospitals between 2016 and 2024 were used if spirometry, single breath gas transfer, and body plethysmography were performed in the same session. GLI reference equations were used to generate z-scores for lung function measures. ICD-10 codes for cardiovascular disease, hypertension, and diabetes were extracted from medical records. We used multi-level (mixed-effects) Cox regression analysis to investigate the association between lung function measurements and incident cardiometabolic disease.

**Results:**

5628 patients were included, 51% were female, with a median age of 62 (IQR 50–70) years. 60% reported a smoking history. Mean follow-up time was 5.7 (SD 2.3) years, during which time 5% received a cardiovascular disease code, 7% a hypertension code, and 3% a diabetes code. A 1-unit increment in TLC z-score was associated with a 12% lower risk of cardiovascular disease (HR: 0.88, 95%CI 0.80–0.97) later in life. The same was seen for FVC (HR: 0.88, 95%CI 0.77–0.99) but not FEV_1_/FVC or DLCO. A larger TLC was also associated with lower risk of myocardial infarction. We found no association of lung function measures with incident hypertension or diabetes.

**Conclusion:**

Lung capacity is a determinant for cardiovascular disease and myocardial infarction, with larger lungs being protective. TLC and FVC should be considered by clinicians along with other factors, when evaluating a person’s risk of cardiovascular disease.

## Background

Cardiovascular diseases are the 2nd leading cause of death in the United Kingdom (UK), accounting for 10% of all deaths in 2023 [1]. Globally, cardiovascular diseases are the leading cause of death [2], with developing countries, like those in Africa, seeing a 50% increase in cardiovascular disease burden over the last 30 years [3]. Therefore, accurate identification of those at risk has never been more important.

The main modifiable risk factors for cardiovascular disease include obesity, dyslipidaemia, hypertension, diabetes, cigarette smoking, and low level of education. However, their contribution to cardiovascular disease risk has declined over recent years, due to the success of public health interventions [4]. This has focused attention on other risk factors, with increasing interest in lung function as a determinant of cardiovascular disease risk [5–9]. In 1983, Kannel et al. published data from the Framingham cohort study showing that forced vital capacity (maximum volume of air forcibly exhaled from the lungs), normalised for height (FVC/height), was a strong independent predictor of cardiovascular disease risk, even after adjustment for known risk factors [10]. Subsequent studies have supported this finding and have suggested that low lung capacity (i.e. FVC), but not airflow obstruction assessed by the FEV_1_/FVC, is associated with incident cardiovascular disease [6, 8, 11]. However, the FVC is an imprecise measure of lung capacity as it does not include the residual volume of the lungs. The gold standard measure of lung capacity is the total lung capacity (TLC) assessed by body plethysmography.

To date, no studies have investigated the association between TLC and incident cardiovascular disease. Therefore, it is largely unknown whether the association between FVC and cardiovascular disease is due to having small lungs or is related to other factors that can limit the FVC [12]. To answer this question, we used a large dataset from patients referred for lung function testing at a tertiary care centre to investigate the association of several lung function parameters, including TLC, with incident cardiometabolic diseases.

## Methods

### Study population

We analysed data from adults (18–80 years) referred for lung function testing at Cambridge University Hospitals (CUH) NHS FT, for any referral reason, between the 1st of January 2016 and the 31st of December 2023. Demographic data included age at time of test, sex, height, weight, body mass index (BMI), ethnicity, and smoking history (never or ever). Data were included in this study from the first instance a patient performed spirometry, single breath gas transfer, and body plethysmography in a single testing session, and had acceptable and reproducible lung function measurements according to the most up to date American Thoracic Society/European Respiratory Society (ATS/ERS) standards [13–15]. Data were excluded if the patient had opted out of their data being used for research purposes. Diagnoses/referral reasons were confirmed by cross-checking with international classification of disease (ICD-10) codes in electronic health records. Approval to use the data was provided by the EHR Research and Innovation (ERIN) database data access committee (IRAS: 318784, study reference: A096987).

### Lung function

Spirometry, single breath gas transfer, and body plethysmography were performed on the MasterScreen-PFT-Pro from Vyaire Medical™. Trained respiratory physiologists instructed all tests, and only those equivalent to grades A or B according to ATS/ERS quality standards were included in our analysis [13–15]. Spirometry measures included the FVC (L), the forced expiratory volume in 1 s (FEV_1_, L), and the FEV_1_/FVC ratio, single breath gas transfer, the diffusing capacity for carbon monoxide corrected for haemoglobin (DLCO, mmol/minute/kPa), and plethysmography the TLC (L). Raw values were converted into z-scores using GLI race specific reference equations for people of European ancestry [16–18]. The lower limit of normal (LLN) for lung function measures was defined as a z-score of -1.645 or as the 5th percentile of a healthy non-smoking population.

### Cardiometabolic diseases

ICD-10 codes for cardiometabolic diseases were extracted from electronic health records held at CUH and linked with patient data in our study database. Codes were collected from encounters, problem lists, and medical histories. We considered a patient to have cardiovascular disease if the ICD-10 codes I20-I25 for ischaemic heart disease, I30-I52 for other forms of heart disease, I60-I69 for cerebrovascular diseases, or I70-I79 for diseases of arteries, arterioles and capillaries were noted in their medical record; hypertension if the ICD-10 code I10 for essential (primary hypertension) was noted in their medical record; and diabetes if the ICD-10 code E10 for type 1 diabetes mellitus or E11 for type 2 diabetes were noted in their medical record. Prevalent cardiometabolic disease was defined as an ICD-10 code for one these conditions noted prior to the date of lung function testing. Incident disease was defined if the ICD-10 code was noted after the date of lung function testing.

### Statistical analysis

We assessed the normality of continuous data by inspecting Q-Q plots and histograms. We reported the mean with standard deviation (SD) for normally distributed data, and the median with interquartile range (IQR) for data not normally distributed. We reported categorical data as the number and percentage in each group. We used descriptive statistics to summarise the baseline characteristics of the study population overall, stratified by the presence of cardiometabolic disease (cardiovascular disease, hypertension, and diabetes), and by referral reason/diagnosis. Using follow-up data, we calculated the incidence rates of cardiometabolic diseases per 1000-person years. We calculated incidence rates for the overall population, and stratified results by the presence of lung function abnormalities and referral reason/diagnosis. We investigated differences in the characteristics of those with vs. without incident cardiometabolic disease using the independent samples t-test for continuous data and the chi-squared test for binary data.

To estimate the association between baseline lung function and incident cardiometabolic disease, we performed multi-level (mixed effects) Cox proportional hazards regression analysis and reported the hazard ratio (HR) with 95% confidence intervals (95%CI). We fitted the models with referral reason/diagnosis as a random intercept, to account for clustering by diagnosis, and lung function measure as a random slope, to average the association of lung function with incident cardiometabolic disease across the different diagnoses. Hazard ratios were presented per 1-unit increment in z-score for each lung function measure. We adjusted our models for age, sex, BMI, and smoking history (ever/never), which are factors we identified a priori as being associated with both lung function and cardiometabolic disease risk [5, 19, 20]. For associations with FVC, we additionally adjusted for the FEV_1_/FVC, as airflow obstruction can lead to underestimation of FVC due to dynamic compression and airway collapse on forced exhalation [21]. For associations with cardiovascular disease, we performed sensitivity analyses for TLC and FVC excluding those with hypertension or diabetes at baseline. To account for potential residual confounding due to undiagnosed cardiovascular disease present before lung function measurement, we also performed a sensitivity analysis excluding those diagnosed with cardiovascular disease within 12-months of lung function testing. We considered results significant if the p-value was less than 0.05. Analyses were performed using Stata version 18 (Stata Corp., College Station, TX, USA).

## Results

**Fig. 1:**
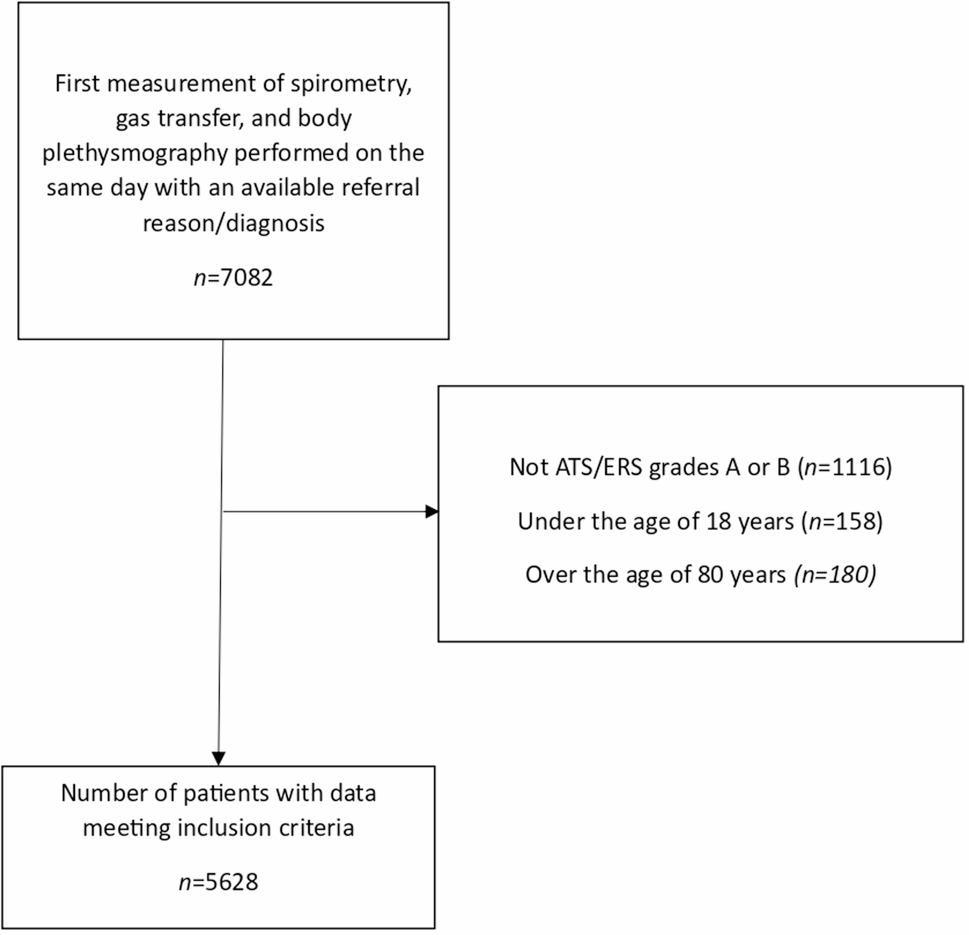
Study flow diagram

Between the 1st of January 2016 and the 31st of December 2023, 7082 first patient visits were recorded in the lung function database at CUH, where patients performed spirometry, single breath gas transfer, and body plethysmography in the same session and had an available referral reason/diagnosis (Fig. 1). Of these, 1116 sessions were excluded due to not meeting ATS/ERS quality grades A or B, 338 were excluded as patients were under the age of 18 or above the age of 80 years at the time of testing. Data from 5628 patients were included in the study. Table 1 presents the characteristics of the study population overall and stratified by the presence of cardiometabolic disease at baseline. Females made up 51% (2851 of 5628) of the study population. Median age was 62 (IQR 50–70) years, mean BMI was 28.4 (SD 6.4), 60% (3393 of 5628) reported a smoking history, and 95% (5348 of 5628) were of European ancestry. The least common referral reason/diagnosis was bronchiectasis (3%, 193 of 5628), and the most common was COPD (25%, 1417 of 5628), followed by asthma (16%, 918 of 5628), and unexplained breathlessness (15%, 833 of 5628). At baseline, 35% (1949 of 5628) of the study population had an FEV_1_/FVC less than the LLN, 14% (804 of 5628) had an FVC less than the LLN, 11% (613 of 5628) had a TLC less than the LLN, and 36% (2002 of 5628) had a DLCO less than the LLN. Lung function impairment varied according to expected disease pathophysiology (Table 2). The prevalence of cardiovascular disease at baseline was 3% (181 of 5628), hypertension 11% (567 of 5628), and diabetes 4% (226 of 5628) (Table 1).

**Table 1:** Characteristics of study participants stratified by presence of cardiometabolic disease at baseline

	Overall (*n* = 5628)	No cardiometabolic disease (*n* = 4882)	Cardiovascular disease (*n* = 181)	Hypertension (*n* = 567)	Diabetes (*n* = 226)
Female, n (%)	2851 (51%)	2443 (50%)	93 (52%)	316 (56%)	119 (53%)
Age, years, median (IQR)	62 (50–70)	62 (50–70)	62 (51–70)	61 (50–70)	62 (51–70)
BMI, mean (SD)	28.4 (6.4)	28.5 (6.4)	28.4 (6.1)	28.1 (6.3)	28.2 (6.0)
Ever smoke, n (%)	3393 (60%)	2962 (61%)	108 (60%)	317 (56%)	140 (61%)
White, n (%)	5348 (95%)	4649 (95%)	174 (96%)	529 (93%)	212 (94%)
Diagnosis/referral reason					
Asthma	918 (16%)	800 (16%)	28 (16%)	91 (16%)	24 (11%)
COPD	1417 (25%)	1240 (25%)	44 (24%)	137 (24%)	51 (23%)
ILD	441 (8%)	378 (8%)	16 (9%)	47 (8%)	21 (9%)
Unexplained shortness of breath	833 (15%)	696 (15%)	36 (20%)	103 (18%)	38 (17%)
Vasculitis	253 (5%)	220 (5%)	7 (4%)	23 (4%)	12 (5%)
Sarcoidosis	416 (7%)	360 (7%)	9 (5%)	43 (8%)	19 (8%)
Bronchiectasis	193 (3%)	168 (3%)	5 (3%)	18 (3%)	9 (4%)
Bone marrow transplant assessment	560 (10%)	509 (10%)	12 (7%)	37 (7%)	21 (9%)
Immunodeficiency	309 (6%)	272 (6%)	10 (6%)	32 (6%)	14 (6%)
Rheumatoid lung disease	288 (5%)	239 (5%)	14 (8%)	36 (6%)	17 (8%)
Lung Function					
FVC z score, mean (SD)	-0.40 (1.18)	-0.41 (1.18)	-0.42 (1.24)	-0.37 (1.17)	-0.37 (1.19)
FVC < LLN	804 (14%)	707 (15%)	25 (14%)	76 (13%)	30 (13%)
FEV1/FVC z-score, mean (SD)	-1.17 (1.84)	-1.18 (1.85)	-1.13 (1.73)	-1.00 (1.82)	-0.88 (1.55)
FEV1/FVC < LLN n (%)	1949 (35%)	1713 (35%)	68 (38%)	179 (32%)	56 (25%)
TLC z-score, mean (SD)	0.12 (1.39)	0.13 (1.40)	-0.02 (1.40)	0.05 (1.26)	-0.03 (1.38)
TLC < LLN, n (%)	613 (11%)	534 (11%)	23 (13%)	59 (10%)	28 (12%)
DLCO z score, mean (SD)	-1.32 (1.91)	-1.34 (1.92)	-1.16 (1.76)	-1.24 (1.84)	-1.31 (1.84)
DLCO < LLN	2002 (36%)	1753 (36%)	65 (36%)	194 (34%)	74 (33%)

Cardiovascular disease classified as having the ICD-10 codes I20-I25 for ischaemic heart disease, or I30-I52 for other forms of heart disease in the medical record at the time of lung function testing, or I60-I69 for cerebrovascular diseases at the time of lung function testing, or I70-I79 for diseases of arteries, arterioles, and capillaries at the time of lung function testing. Hypertension classified as having the ICD-10 code I10 for essential (primary hypertension) in the medical record at the time of lung function testing. Diabetes classified as having the ICD-10 code E10 for Type 1 diabetes mellitus, or E11 for Type 2 diabetes mellitus in the medical record at the time of lung function testing. FVC: Forced vital capacity; FEV1: Forced expiratory volume in 1 s; TLC: Total lung capacity: DLCO: total lung gas transfer; LLN: Lower limit of normal; SD: standard deviation; IQR: interquartile range; CVD: cardiovascular disease. For spirometry, z -scores and LLN calculated using race-specific reference equations from the global lung function initiative (GLI) [16]. For TLC, and DLCO, z-scores and LLN calculated using reference equations for white Europeans from the GLI [17, 18]

**Table 2:** Baseline characteristics stratified by referral reason/diagnosis

	Asthma (*n* = 918)	COPD (*n* = 1417)	ILD (*n* = 441)	Unexplained shortness of breath (*n* = 833)	Vasculitis (*n* = 253)	Sarcoidosis (*n* = 416)	Bronchiectasis (*n* = 193)	Bone marrow transplant assessment (*n* = 560)	Immunodeficiency (*n* = 309)	Rheumatoid lung disease (*n* = 288)
Female, n (%)	546 (60%)	582 (41%)	204 (46%)	426 (51%)	141 (56%)	174 (42%)	129 (67%)	211 (38%)	189 (61%)	249 (87%)
Age, years, median (IQR)	55 (44–66)	66 (57–72)	70 (62–75)	63 (52–71)	59 (49–70)	54 (45–64)	66 (57–74)	58 (48–65)	53 (37–67)	58 (48–68)
BMI, mean (SD)	29.4 (6.6)	27.2 (6.3)	28.9 (6.0)	30.0 (6.6)	29.3 (6.4)	29.0 (5.6)	27.2 (6.4)	27.4 (5.4)	27.7 (6.4)	27.8 (6.7)
Ever smoke, n (%)	456 (50%)	1308 (92%)	259 (59%)	466 (56%)	112 (44%)	185 (45%)	98 (51%)	241 (43%)	144 (46%)	124 (43%)
White, n (%)	845 (92%)	1398 (99%)	421 (96%)	799 (96%)	236 (93%)	379 (91%)	181 (94%)	533 (95%)	300 (97%)	256 (89%)
Lung Function										
FVC z score, mean (SD)	-0.40 (1.14)	-0.49 (1.20)	-0.70 (1.22)	-0.47 (1.17)	-0.50 (1.22)	-0.21 (0.99)	-0.36 (1.15)	-0.07 (1.18)	-0.22 (1.16)	-0.47 (1.20)
FVC < LLN	121 (13%)	234 (16%)	89 (20%)	126 (15%)	46 (18%)	31 (8%)	22 (11%)	54 (10%)	34 (11%)	47 (16%)
FEV1/FVC z-score, mean (SD)	-1.16 (1.55)	-2.55 (1.88)	-0.12 (1.39)	-0.71 (1.75)	-0.91 (1.58)	-0.61 (1.26)	-1.11 (1.41)	-0.48 (1.32)	-0.58 (1.92)	-0.29 (1.33)
FEV1/FVC < LLN n (%)	336 (36%)	977 (69%)	46 (10%)	189 (22%)	71 (28%)	75 (18%)	69 (36%)	91 (16%)	55 (18%)	40 (14%)
TLC z-score, mean (SD)	0.04 (1.14)	1.05 (1.42)	-0.86 (1.39)	-0.18 (1.11)	-0.42 (1.32)	-0.25 (1.09)	0.25 (1.10)	-0.01 (1.19)	-0.09 (1.21)	-0.40 (1.36)
TLC < LLN, n (%)	72 (8%)	44 (3%)	152 (35%)	90 (11%)	47 (19%)	52 (13%)	11 (6%)	52 (9%)	35 (11%)	58 (20%)
DLCO z score, mean (SD)	-0.21 (1.34)	-2.83 (2.13)	-2.02 (1.71)	-0.75 (1.43)	-0.94 (1.54)	-0.56 (1.40)	-0.61 (1.37)	-0.97 (1.25)	-0.69 (1.41)	-1.21 (1.72)
DLCO < LLN	122 (13%)	962 (68%)	247 (56%)	185 (22%)	77 (30%)	73 (18%)	29 (15%)	143 (25%)	64 (21%)	100 (35%)

FVC: Forced vital capacity; FEV1: Forced expiratory volume in 1 s; TLC: Total lung capacity: DLCO: total lung gas transfer; LLN: Lower limit of normal; SD: standard deviation; IQR: interquartile range; ILD: interstitial lung disease. For spirometry, z -scores and LLN calculated using race-specific reference equations from the global lung function initiative (GLI) [16]. For TLC, and DLCO, z-scores and LLN calculated using reference equations for white Europeans from the GLI [17, 18]

### Cardiovascular disease

**Fig. 2:**
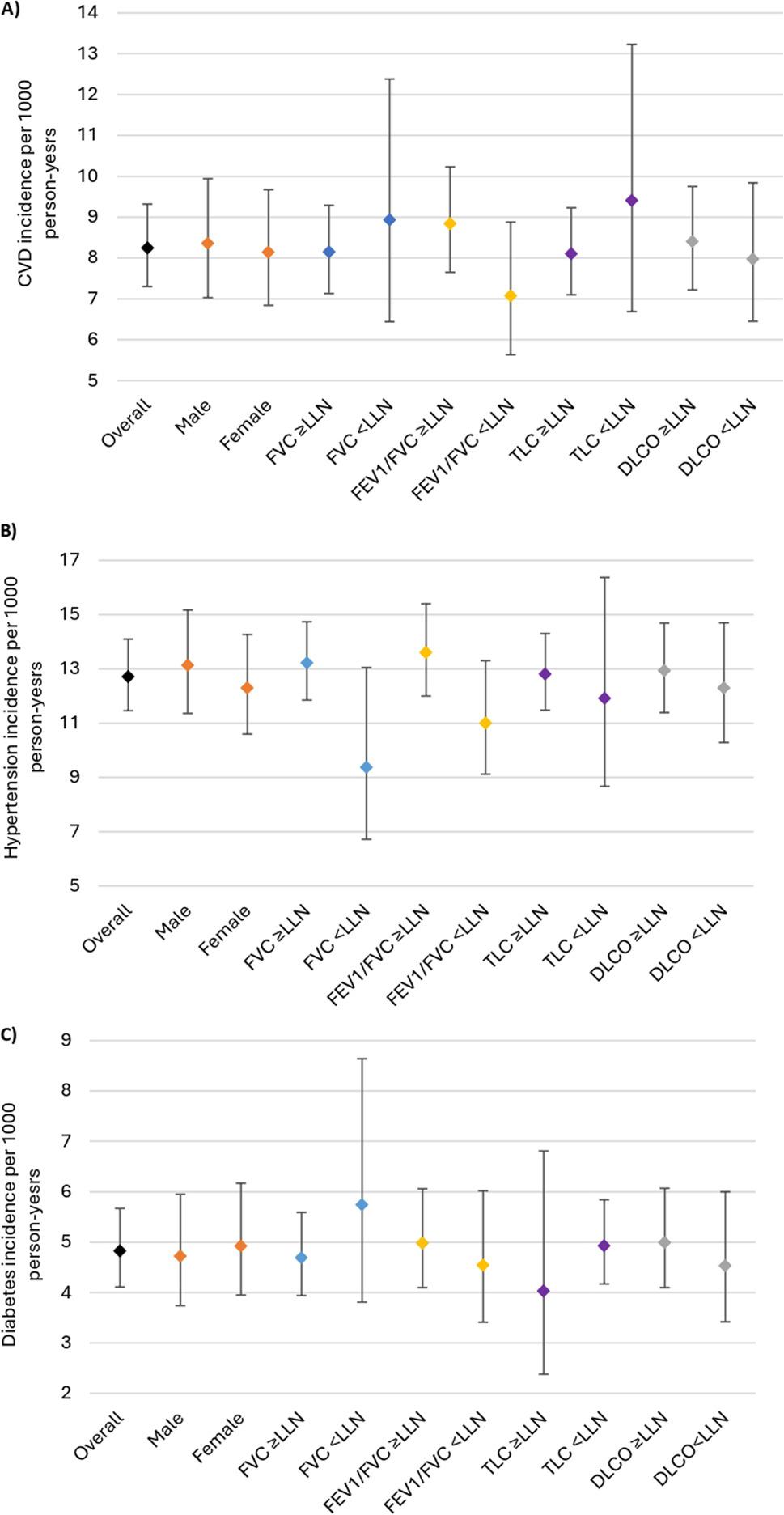
Incidence of (**A**) cardiovascular disease, (**B**) hypertension, and (**C**) Diabetes stratified by sex and lung function

**Fig. 3:**
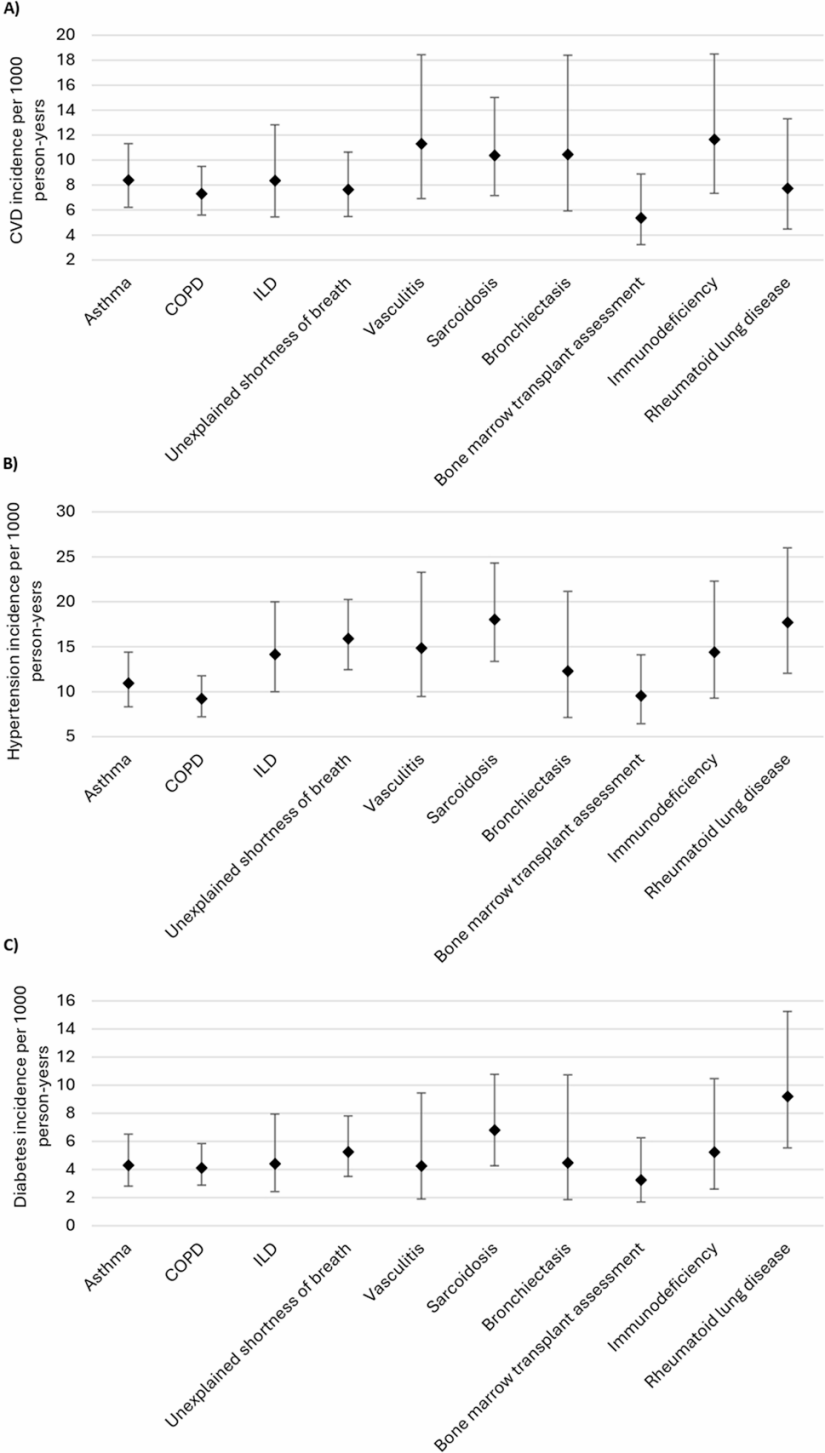
Incidence of (**A**) cardiovascular disease, (**B**) hypertension, and (**C**) Diabetes stratified by referral reason/diagnosis

97% of the patients (5447 of 5628) did not have cardiovascular disease at baseline. Of these, 5% (256 of 5447) developed cardiovascular disease over a mean follow-up of 5.7 (SD 2.3) years. Incident myocardial infarction was noted in 113 of 256 (44%), angina in 24 of 256 (9%), chronic heart disease in 39 of 256 (15%), stroke 71 of 256 (28%), and peripheral vascular disease in 9 of 256 (4%). Figure 2a shows the incidence rates of cardiovascular disease per 1000 person-years overall and stratified by lung function. Overall, the incidence rate of cardiovascular disease was 8.25 (95%CI, 7.30–9.32) per 1000 person-years and was similar for males and females. Compared to those with a TLC ≥ LLN (8.10, 95%CI 7.10–9.23 per 1000 person years), the incidence rate of cardiovascular disease was higher in patients with a TLC < LLN (9.41, 95%CI 6.69–13.23 per 1000 person years). Incidence of cardiovascular disease was also higher among those with a FEV_1_/FVC ≥ LLN (8.84 95%CI 7.65–10.23 per 1000 person-years), compared to those with a FEV_1_/FVC < LLN (7.07, 95%CI 5.63–8.88 per 1000 person-years). There was no difference in the incidence of cardiovascular disease regardless of whether the DLCO was normal or not. When stratifying by referral reason/diagnosis (Fig. 3a), incidence of cardiovascular disease was highest in vasculitis, sarcoidosis, bronchiectasis, and immunodeficiency, relative to other referral reasons/diagnoses.

At baseline (Table 3), compared to those who did not develop cardiovascular disease, those with incident disease had a lower TLC (mean TLC z-score: -0.13 vs. 0.14, *p* = 0.003), were less likely to have ever smoked (55% vs. 61%, *p* = 0.06), and were less likely to have airflow obstruction (29% vs. 35%, *p* = 0.05). After adjusting for potential confounding, the results of the Cox regression analyses showed that per 1-unit increment in TLC z-score (larger lung capacity), risk of cardiovascular disease reduced by 12% (HR: 0.88, 95%CI 0.80–0.97). The same was seen for FVC (HR: 0.88, 95%CI 0.77–0.99) (Table 4). Results for TLC (HR: 0.88, 95%CI 0.79–0.98) and FVC (HR: 0.85, 95%CI 0.72–0.99) did not materially change when those with diabetes or hypertension at baseline were excluded. Nor did results materially change for TLC (HR: 0.86, 95%CI 0.77–0.95) or FVC (HR: 0.89, 95%CI 0.79–1.02) when those with incident cardiovascular disease within 12 months of lung function (36 of 256) were excluded. There was no association of FEV_1_/FVC or DLCO with incident cardiovascular disease. In a secondary analysis, we found that a larger TLC was associated with a lower risk of incident myocardial infarction (HR: 0.87, 95%CI 0.76–0.99, *p* = 0.049), the results for FVC suggested a similar association but did not reach statistical significance (HR: 0.89, 95%CI 0.76–1.06, *p* = 0.195).

**Table 3:** Characteristics of those who did vs did not develop cardiovascular disease, hypertension, and diabetes

	Incident Cardiovascular disease			Incident Hypertension			Incident Diabetes		
	Yes (*n* = 256)	No (*n* = 5191)	*p*-value	Yes (*n* = 358)	No (*n* = 4703)	*p*-value	Yes (*n* = 149)	No (*n* = 5253)	*p*-value
Female, n (%)	128 (50%)	2360 (51%)	0.836	174 (49%)	2361 (50%)	0.560	77 (52%)	2655 (51%)	0.785
Age, years, median (IQR)	61 (50–69)	62 (50–70)	0.401	60.5 (48–70)	62 (50–70)	0.182	61 (49–70)	61 (50–70)	0.964
BMI, mean (SD)	28.0 (6.7)	28.4 (6.4)	0.294	28.8 (6.71)	28.4 (6.34)	0.314	28.5 (6.29)	28.4 (6.38)	0.882
Ever smoke, n (%)	140 (55%)	3145 (61%)	0.060	203 (57%)	2873 (61%)	0.101	85 (57%)	3168 (61%)	0.423
White, n (%)	241 (94%)	4933 (95%)	0.244	337 (94%)	4482 (95%)	0.311	139 (93%)	4997 (95%)	0.217
Lung Function									
FVC z score, mean (SD)	-0.45 (1.17)	-0.40 (1.18)	0.515	-0.27 (1.04)	-0.42 (1.19)	0.023	-0.39 (1.17)	-0.41 (1.18)	0.904
FVC < LLN	36 (14%)	743 (14%)	0.911	35 (10%)	693 (15%)	0.010	23 (15%)	751 (14%)	0.695
FEV1/FVC z-score, mean (SD)	-1.06 (1.54)	-1.18 (1.86)	0.352	-1.06 (1.38)	-1.20 (1.86)	0.185	-1.06 (1.68)	-1.18 (1.85)	0.418
FEV1/FVC < LLN n (%)	74 (29%)	1811 (35%)	0.050	107 (30%)	1667 (36%)	0.034	48 (32%)	1847 (35%)	0.457
TLC z-score, mean (SD)	-0.13 (1.31)	0.14 (1.39)	0.003	0.04 (1.09)	0.13 (1.43)	0.271	0.13 (1.39)	0.15 (1.34)	0.599
TLC < LLN, n (%)	36 (14%)	556 (11%)	0.201	38 (11%)	516 (11%)	0.835	18 (12%)	567 (11%)	0.568
DLCO z-score, mean (SD)	-1.31 (1.95)	-1.32 (1.92)	0.974	-1.18 (1.76)	-1.34 (1.93)	0.147	-1.11 (1.84)	-1.33 (1.92)	0.174
DLCO < LLN	86 (33%)	1851 (36%)	0.501	121 (34%)	1687 (36%)	0.430	49 (32%)	1879 (36%)	0.469

Incident cardiovascular disease classified as having the ICD-10 codes I20-I25 for ischaemic heart disease, or I30-I52 for other forms of heart disease in the medical record after the date of lung function testing, or I60-I69 for cerebrovascular diseases in the medical record after the date of lung function testing, or I70-I79 for diseases of arteries, arterioles and capillaries in the medical record after the date of lung function testing. incident hypertension classified as having the ICD-10 code I10 for essential (primary hypertension) in the medical record after the date of lung function testing. incident diabetes classified as having the ICD-10 code E10 for type 1 diabetes mellitus, or E11 for type 2 diabetes mellitus in the medical record after the date of lung function testing. FVC: forced vital capacity; FEV1: forced expiratory volume in 1 s; TLC: total lung capacity: DLCO: total lung gas transfer; LLN: lower limit of normal; SD: standard deviation; IQR: interquartile range. For spirometry, z -scores and LLN calculated using race-specific reference equations from the global lung function initiative (GLI) [16]. For TLC, and DLCO, z-scores and LLN calculated using reference equations for white Europeans from the GLI [17, 18]. For continuous variables, differences between those with vs. without incident disease calculated using the independent samples t-test. For binary variables, differences in proportions for those with vs. without incident disease calculated using the chi-squared test

**Table 4:** Association of lung function with incident cardiovascular disease

	Total *n*	CVD *n* (%)	Unadjusted HR (95%CI)	*p*-value	Adjusted HR (95%CI)	*p*-value
FVC z-score	5447	256 (5%)	0.93 (0.84–1.04)	0.187	0.88 (0.77–0.99)	0.034
FEV1/FVC z-score	5447	256 (5%)	1.04 (0.97–1.13)	0.208	1.03 (0.96–1.12)	0.340
TLC z-score	5447	256 (5%)	0.88 (0.81–0.96)	0.008	0.88 (0.80–0.97)	0.008
DLCO z-score	5447	256 (5%)	1.00 (0.95–1.05)	0.985	0.97 (0.90–1.04)	0.397

Incident cardiovascular disease classified as having the ICD-10 codes I20-I25 for ischaemic heart disease, or I30-I52 for other forms of heart disease in the medical record after the date of lung function testing, or I60-I69 for cerebrovascular diseases in the medical record after the date of lung function testing, or I70-I79 for diseases of arteries, arterioles and capillaries in the medical record after the date of lung function testing. To estimate the association between a 1-unit increase in lung function z-score at baseline and cardiovascular disease at follow-up, we performed multi-level (mixed effects) cox proportional hazards regression analysis, and reported the hazard ratio (HR) with 95% confidence intervals (95%CI). We fitted the models with a random intercept with diagnosis/referral reason as the effect term, to account for clustering by diagnosis/referral reason, and a random slope with lung function z-score as the effect term, to average the association of a 1-unit increase in z-score with incident cardiovascular disease across the different diagnoses/referral reasons. FVC: Forced vital capacity; FEV1: Forced expiratory volume in 1 s; TLC: Total lung capacity: DLCO: total lung gas transfer; CVD: cardiovascular disease. For spirometry, z -scores calculated using race-specific reference equations from the global lung function initiative (GLI) [16]. For TLC, and DLCO, z-scores calculated using reference equations for white Europeans from the GLI [17, 18]

### Hypertension

90% of the patients (5061 of 5628) did not have hypertension at baseline. Of these, 7% (358 of 5061) developed hypertension. Overall, the incidence rate of hypertension was 12.71 (95%CI, 11.46–14.10) per 1000 person-years, it was similar for males and females and was lower for those with lung function abnormalities (Fig. 2b). When stratifying by referral reason/diagnosis (Fig. 3b), incidence of hypertension was highest for sarcoidosis and rheumatoid lung disease, and lowest for asthma, COPD, and bone marrow transplant assessment.

At baseline (Table 3), compared to those who did not develop hypertension, those with incident hypertension were less likely to have an FVC < LLN (10% vs. 15%, *p* = 0.01) and were less likely to have an FEV_1_/FVC < LLN (30% vs. 36%, *p* = 0.034). All other characteristics were similar. The results of the Cox regression analysis showed no association of incident hypertension with any of the lung function parameters (Table 5).

**Table 5:** Association of lung function with incident hypertension

	Total *n*	hypertension *n* (%)	Unadjusted HR (95%CI)	*p*-value	Adjusted HR (95%CI)	*p*-value
FVC z-score	5061	358 (7%)	1.08 (0.99–1.18)	0.097	1.08 (0.99–1.19)	0.086
FEV1/FVC z-score	5061	358 (7%)	1.03 (0.96–1.10)	0.384	1.02 (0.95–1.10)	0.517
TLC z-score	5061	358 (7%)	1.00 (0.92–1.09)	0.982	1.01 (0.93–1.11)	0.721
DLCO z-score	5061	358 (7%)	1.01 (0.97–1.06)	0.419	0.98 (0.92–1.05)	0.621

Incident hypertension classified as having the ICD-10 code I10 for essential (primary hypertension) in the medical record after the date of lung function testing. To estimate the association between a 1-unit increase in lung function z-score at baseline and hypertension at follow-up, we performed multi-level (mixed effects) cox proportional hazards regression analysis, and reported the hazard ratio (HR) with 95% confidence intervals (95%CI). We fitted the models with a random intercept with diagnosis/referral reason as the effect term, to account for clustering by diagnosis/referral reason, and a random slope with lung function z-score as the effect term, to average the association of a 1-unit increase in z-score with incident hypertension across the different diagnoses/referral reasons. FVC: Forced vital capacity; FEV1: Forced expiratory volume in 1 s; TLC: Total lung capacity: DLCO: total lung gas transfer; CVD: cardiovascular disease. For spirometry, z -scores calculated using race-specific reference equations from the global lung function initiative (GLI) [16]. For TLC, and DLCO, z-scores calculated using reference equations for white Europeans from the GLI [17, 18]

### Diabetes

90% of the patients (5042 of 5628) did not have diabetes at baseline. Of these, 3% (149 of 5042) developed diabetes. Overall, the incidence rate of diabetes was 4.83 (4.11–5.67) per 1000 person-years (Fig. 2c). Incidence was similar for males and females and was higher for those with a low FVC. When stratifying by referral reason/diagnosis (Fig. 3c), incidence was highest in rheumatoid lung disease and lowest in bone marrow transplant assessment.

At baseline (Table 3), the characteristics of those with incident diabetes were similar to those who did not develop diabetes. The results of the Cox regression analysis showed no association of diabetes with any of the lung function parameters (Table 6).

**Table 6:** Association of lung function with incident diabetes

	Total *n*	Diabetes *n* (%)	Unadjusted HR (95%CI)	*p*-value	Adjusted HR (95%CI)	*p*-value
FVC z-score	5402	149 (3%)	0.98 (0.85–1.12)	0.739	0.98 (0.85–1.12)	0.740
FEV1/FVC z-score	5402	149 (3%)	1.06 (0.96–1.16)	0.258	1.05 (0.95–1.16)	0.333
TLC z-score	5402	149 (3%)	1.03 (0.91–1.16)	0.582	1.05 (0.92–1.19)	0.454
DLCO z-score	5402	149 (3%)	1.03 (0.96–1.10)	0.372	1.04 (0.95–1.15)	0.367

Incident diabetes classified as having the ICD-10 code E10 for Type 1 diabetes mellitus, or E11 for Type 2 diabetes mellitus in the medical record after the date of lung function testing. To estimate the association between a 1-unit increase in lung function z-score at baseline and diabetes at follow-up, we performed multi-level (mixed effects) cox proportional hazards regression analysis, and reported the hazard ratio (HR) with 95% confidence intervals (95%CI). We fitted the models with a random intercept with diagnosis/referral reason as the effect term, to account for clustering by diagnosis/referral reason, and a random slope with lung function z-score as the effect term, to average the association of a 1-unit increase in z-score with incident diabetes across the different diagnoses/referral reasons. FVC: Forced vital capacity; FEV1: Forced expiratory volume in 1 s; TLC: Total lung capacity: DLCO: total lung gas transfer; CVD: cardiovascular disease. For spirometry, z -scores calculated using race-specific reference equations from the global lung function initiative (GLI) [16]. For TLC, and DLCO, z-scores calculated using reference equations for White Europeans from the GLI [17, 18]

## Discussion

To the best of our knowledge, our study is the first to investigate the association between lung function and incident cardiometabolic disease in a large patient cohort. We have shown that a greater lung capacity, assessed by either TLC or FVC, is associated with reduced risk of incident cardiovascular disease and acute myocardial infarction. We found no association between airflow obstruction or reduced diffusing capacity with incident cardiovascular disease. We also found no association of incident hypertension or diabetes with lung function parameters.

In the present study, over a mean follow-up of 5.7 years, we found that the incidence rate of cardiovascular disease was higher for patients with low TLC compared to those with normal values. Furthermore, we found that per 1-unit increment in baseline TLC z-score, risk of cardiovascular disease reduced by 12%, as did the risk of acute myocardial infarction. There are no studies available for direct comparison. However, a study by Chang et al. in 440 ambulatory heart failure patients [22] found that TLC was reduced at baseline and that, per 1-SD increase in TLC, the risk of cardiovascular mortality decreased by 44%. It is difficult to compare these results to our study, where patient populations were diverse, as reduced TLC is well documented in heart failure and has been shown to be associated with pulmonary congestion [23]. A better comparison is a study by Amaral et al. [7], which used population-based data from 108 participants from the London site of the multinational Burden of Obstructive Lung Disease (BOLD) study. They investigated the association between TLC assessed by plethysmography and pulse wave velocity, a measure of arterial stiffness and cardiovascular disease risk. They found an inverse association, with greater TLC associated with lower arterial stiffness, supporting our findings that low TLC is a risk factor for cardiovascular disease.

Like for TLC, we found that a higher FVC was also associated with lower risk of incident cardiovascular disease. This association is well documented in the literature [10, 24–26]. In a similar analysis, Li et al. [26] used data from the UK Biobank study and fond that a higher FVC was associated with lower risk of incident coronary heart disease and stroke classified using ICD-10 codes. Another recent publication by Janson et al. [6] further examined the association between lung function and incident heart disease using data from 6000 participants of the multinational BOLD study. Over 9.5 years of follow-up, they found that a higher baseline FVC was associated with lower odds of heart disease. Interestingly, like our study, Janson et al. also found no association for airflow obstruction assessed using the FEV_1_/FVC [6]. This is not the first time this observation has been made. Johnston et al. [11], using data from a cohort of 14,681 US adults, found that after adjustment for confounding, the association of airflow obstruction with incident cardiovascular disease approached the null. Together with our findings, this suggests that the risk of incident cardiovascular disease among people with obstructive lung disease, such as COPD, as documented in the literature is likely driven by shared risk factors (e.g. smoking) or short-term exposures (e.g. viral infections) leading to acute cardiovascular events.

We previously put forward the term small lung syndrome, to describe individuals with a low FVC, who are at increased risk of cardiovascular morbidity and mortality [27]. As we found the magnitude of the effect to be similar between TLC and FVC, our results suggest that it is the dynamic volume measured by the FVC that is associated with cardiovascular disease risk. However, the mechanisms explaining this association are not fully understood. A possible mechanism relates to increased systemic inflammation. A study of 5064 healthy adult males by Engström et al. [8], found that those in the lowest quartile for FVC had greater incidence of cardiovascular mortality and myocardial infarction over 18.4 years of follow-up. They also had higher circulating levels of inflammatory plasma proteins. This was supported by another study using data from the National Health and Nutrition Examination Survey (NHANES), which found that FVC was inversely associated with C-reactive protein (CRP) [28]. Interestingly, in our study, incidence of cardiometabolic disease was generally higher in diseases associated with autoimmune inflammation. However, other studies have found no evidence supporting the hypothesis of systemic inflammation [7]. It is also possible that the association between TLC and cardiovascular disease relates to sub-optimal organ development in utero or early childhood, with further research needed in this area.

We found no association between lung function parameters and incident hypertension or diabetes. This does not support what has been previously shown in the literature, where FVC has been found to have a multidirectional association with these conditions [6, 29–32]. A possible explanation is that we defined hypertension according to ICD-10 code rather than through direct measurement of blood pressure. This could introduce bias, as the medical record entry was likely made during a doctor’s appointment or hospital visit, where blood pressure measurements, particularly if no more than one measurement was made, could be raised due to anxiety, or an underlying medical condition. Furthermore, it is possible that patients had hypertension that was not yet diagnosed, as blood pressure was only checked if clinically indicated and not measured in all patients. Similarly, for diabetes, we did not directly measure blood glucose or HbA1C, increasing risk of measurement error.

The strengths of our study include its large sample size, and measurement of detailed lung physiology according to strict acceptability and reproducibility criteria. Furthermore, for cardiovascular disease, the use of ICD-10 codes to confirm diagnoses reduced risk of bias that occurs when disease is self-reported. Our study is not without limitations, firstly, as we mentioned above, the use of ICD-10 codes for diabetes and hypertension increases the risk of measurement bias impacting associations. Furthermore, as this was a hospital-based population, it is difficult to extrapolate our findings to the general population, where levels of lung function impairment and incidence of disease may differ. It is important that large population-based cohorts replicate our findings.

## Conclusions

We have shown that lung capacity is a risk factor for cardiovascular disease and myocardial infarction, with larger lungs being protective. TLC and FVC should be considered by clinicians, along with other factors, when evaluating a person’s risk of cardiovascular disease.

## Data Availability

As this is data from patients from the National Health Service, data is not freely available. Data can be shared with researchers with a full or honorary contract at Cambridge University Hospitals NHS FT. Requesting researchers will be required to submit an analysis plan to Dr Ben Knox-Brown, b.knox-brown20@imperial.ac.uk.

## Abbreviations

TLC: total lung capacity
GLI: Global Lung Function Initiative
TLC: Total lung capacity
FEV1: Forced expiratory volume in 1 s
FVC: Forced vital capacity
DLCO: Diffusing capacity of carbon monoxide
LLN: Lower limit of normal
HR: Hazard ratio
95%CI: 95% confidence interval
SD: Standard deviation
IQR: Interquartile range
COPD: chronic obstructive pulmonary disease
ILD: Interstitial lung disease
ICD: International classification of disease
BMI: Body mass index
